# SIRT1 in Atherosclerosis: Integrative Control of Vascular Metabolism, Inflammation and Aging

**DOI:** 10.3390/ijms27073031

**Published:** 2026-03-26

**Authors:** Yingxuan Chang, Le Li, Hongmei Yue

**Affiliations:** Department of Respiratory Medicine, The First Hospital of Lanzhou University, Lanzhou 730000, China; changyx0019@163.com (Y.C.); c9422880515@gmail.com (L.L.)

**Keywords:** SIRT1, atherosclerosis, inflammation, oxidative stress, macrophage polarization, vascular smooth muscle cells, NAD^+^ metabolism, precision cardiology

## Abstract

Atherosclerosis is a chronic inflammatory and metabolic disease driven by endothelial dysfunction, immune activation, vascular smooth muscle cell remodeling and aging-associated mitochondrial decline. Although lipid lowering remains the cornerstone of therapy, substantial residual inflammatory risk persists, highlighting the need for integrative regulatory targets. Sirtuin 1 (SIRT1), a NAD^+^-dependent deacetylase, has emerged as a central metabolic sensor linking energy availability to transcriptional control of inflammation, oxidative stress, mitochondrial biogenesis and cellular senescence. Experimental studies across endothelial cells, macrophages and vascular smooth muscle cells consistently demonstrate that SIRT1 activation preserves nitric oxide bioavailability, suppresses ROS-dependent inflammasome signaling, modulates macrophage polarization, inhibits ferroptosis and maintains mitochondrial integrity. These cell-type-specific effects converge to reduce plaque progression and enhance fibrous cap stability in preclinical models. However, SIRT1 activity is hierarchically regulated by AMPK signaling and NAD^+^ availability and is influenced by aging, metabolic dysfunction and environmental stressors, underscoring its context-dependent function. Despite promising mechanistic data, clinical translation remains limited, suggesting that precision modulation strategies may be required. This review synthesizes current evidence and proposes that SIRT1 functions as a metabolic–inflammatory integrator within the atherosclerotic arterial wall, representing a potential but context-sensitive target for future cardiovascular therapies.

## 1. Introduction

Atherosclerosis is no longer conceptualized as a passive lipid-storage disorder but as a dynamic maladaptive remodeling process driven by chronic metabolic stress and unresolved inflammation [1,2,3,4]. Although LDL cholesterol is causally implicated in disease initiation, lipid lowering alone fails to fully abolish cardiovascular risk, underscoring the importance of additional regulatory layers that integrate metabolic signals with vascular inflammation [1,5,6]. Recent systems-level analyses have emphasized that lesion progression reflects coordinated dysfunction across endothelial cells, macrophages, vascular smooth muscle cells (VSMCs), and immune networks, all operating within a metabolically constrained environment [1,7,8].

Within this framework, Sirtuin 1 (SIRT1) emerges as a central metabolic integrator. As a NAD^+^-dependent deacetylase, SIRT1 directly couples cellular energy availability to transcriptional reprogramming and stress adaptation [1,2,9,10]. Its enzymatic activity is intrinsically linked to NAD^+^ levels, which decline with aging and metabolic disease, two dominant risk factors for atherosclerosis [2,11]. Through deacetylation of NF-κB, p53, FOXO transcription factors, PGC-1α, eNOS, and HIF-1α, SIRT1 coordinates inflammatory tone, mitochondrial biogenesis, oxidative stress responses, autophagy, and cellular senescence [10,12,13,14].

The central question, however, is not whether SIRT1 influences vascular biology; this is well established, but rather how SIRT1 integrates upstream metabolic disturbances with downstream inflammatory cascades, and whether its modulation of this axis can meaningfully reshape plaque biology.

Over the past decade, the conceptual understanding of SIRT1 in atherosclerosis has shifted from that of a classical anti-inflammatory regulator to that of a multicellular stress integration hub. Rather than acting solely as a linear signaling component, SIRT1 operates at the intersection of metabolic sensing, mitochondrial adaptation, inflammatory regulation, and cellular survival across multiple vascular cell types. This integrative function arises from the intrinsic dependence of SIRT1 on NAD^+^ availability, positioning it as a metabolic sensor responsive to cellular energetic state. Upstream regulators including AMPK, oxidative stress, nutrient availability, and circadian rhythms converge on SIRT1 activity, which in turn coordinates downstream transcriptional programs governing inflammation, mitochondrial biogenesis, and cellular senescence. Importantly, these effects occur simultaneously across endothelial cells, macrophages, and vascular smooth muscle cells, allowing SIRT1 to orchestrate a coordinated vascular adaptation to metabolic and inflammatory stress. In this framework, SIRT1 should be conceptualized not merely as a pathway component, but as a network stabilizer that preserves vascular homeostasis.

In this review, relevant articles were identified through searches of PubMed, Web of Science, and Scopus using combinations of the keywords “SIRT1”, “sirtuin”, “atherosclerosis”, and related metabolic or inflammatory pathways. Approximately 300 publications were initially screened, and the most relevant experimental and translational studies were included in this review.

## 2. Endothelial Cells: SIRT1 as a Redox Buffer and Metabolic Effector

### 2.1. The Endothelium as the Initiating Interface

The endothelium functions as the gatekeeper of vascular homeostasis, regulating leukocyte adhesion, nitric oxide production, barrier integrity and thrombogenic balance [1,15,16]. Endothelial dysfunction represents the earliest detectable event in atherogenesis and is characterized by diminished NO bioavailability, increased expression of adhesion molecules, and heightened oxidative stress. Because SIRT1 deacetylates and activates eNOS while simultaneously suppressing NF-κB-mediated inflammatory transcription, it occupies a strategic position in preserving endothelial stability [2,9].

### 2.2. Oxidative Stress and Inflammasome Control

Experimental evidence indicates that endothelial SIRT1 restrains redox amplification loops. In high-fat diet-fed ApoE^−^/^−^ mice, activation of the SIRT1/Nrf2 pathway significantly reduced aortic ROS levels and attenuated NLRP3 inflammasome activation, leading to diminished lesion formation [17]. Parallel in vitro experiments demonstrated that pharmacological inhibition of SIRT1 abolished antioxidant gene induction and restored inflammasome activation, underscoring that SIRT1 is not merely correlated with but mechanistically required for endothelial redox control. Similarly, in acrolein-induced vascular injury models, restoration of SIRT1 expression suppressed pyroptotic signaling and reduced oxidative damage through activation of the SIRT1/Nrf2 axis [18]. These findings reinforce the concept that SIRT1 governs inflammasome suppression not only by reducing ROS production but by actively reprogramming antioxidant transcriptional networks.

### 2.3. Metabolic Hierarchy: AMPK as an Upstream Gatekeeper

Endothelial SIRT1 activation is metabolically contingent. TMAO, a gut microbiota-derived metabolite strongly associated with atherosclerosis, suppresses AMPK phosphorylation and secondarily diminishes SIRT1 activity, resulting in enhanced ROS production and inflammatory gene expression [19]. Restoration of AMPK activity rescues SIRT1 function, indicating that SIRT1 often acts downstream within a broader metabolic sensing cascade. Multi-omics analyses further demonstrate that TMAO suppresses SIRT1 expression in vascular cells and amplifies inflammatory signaling pathways [20]. These observations suggest that endothelial SIRT1 functions as a metabolic effector node, translating upstream nutrient-derived stress signals into inflammatory responses.

### 2.4. Epigenetic Fine-Tuning via Non-Coding RNAs

SIRT1 expression in endothelial cells is further modulated by non-coding RNAs. miR-199a-5p directly targets SIRT1 and exacerbates endothelial injury in both ox-LDL-treated cells and ApoE^−^/^−^ mice [21]. Silencing this microRNA restores SIRT1 levels and improves endothelial repair capacity. Additionally, lincRNA-p21 and HAND2-AS1 regulate SIRT1 through competing endogenous RNA networks that influence lipid handling and plaque formation [22,23]. Thus, endothelial SIRT1 resides at the convergence of metabolic, inflammatory and epigenetic regulation.

## 3. Macrophages: SIRT1 as a Rheostat of Inflammatory Plasticity

### 3.1. Macrophage Centrality in Plaque Evolution

Following endothelial activation, monocytes infiltrate the intima and differentiate into macrophages [24,25]. These cells internalize modified lipoproteins to form foam cells and secrete cytokines that perpetuate inflammation [1,26]. The balance between pro-inflammatory M1 and anti-inflammatory M2 phenotypes strongly influences plaque trajectory.

### 3.2. Genetic Evidence of Causality

Macrophage-specific deletion of SIRT1 in ApoE^−^/^−^ mice results in significantly increased lesion burden and enhanced expression of M1-associated cytokines [19,27]. Mechanistically, loss of SIRT1 disrupts the TIMP3/ADAM17 axis, promoting TNF-α shedding and inflammatory amplification [19]. This genetic evidence establishes SIRT1 as a direct regulator of macrophage inflammatory programming rather than a secondary marker.

### 3.3. Pharmacological Convergence on SIRT1

Pharmacological studies reveal a recurring pattern: diverse agents converge upon SIRT1 to recalibrate macrophage phenotype. In high-fat diet-fed ApoE^−^/^−^ mice, Nao-Xin-Qing tablets reduced plaque burden while promoting M2 polarization through activation of the AMPKα/SIRT1/PPARγ pathway [11]. Importantly, suppression of AMPK eliminated SIRT1 activation and reversed macrophage repolarization, demonstrating metabolic dependence [22,27]. Dihydromyricetin similarly inhibited M1 polarization in ApoE-deficient mice and macrophage cultures by relieving miR-9-mediated repression of SIRT1 and suppressing NF-κB signaling [28]. Panax notoginseng saponins improved lipid metabolism and attenuated vascular injury via SIRT1/PPARγ signaling in lupus-associated atherosclerosis models [29], illustrating that SIRT1 integrates inflammatory and metabolic signals. Vitamin D supplementation has also been shown to attenuate macrophage-driven inflammation via SIRT1/mTORC2 modulation [8], further supporting the idea that SIRT1 functions as a nodal inflammatory rheostat responsive to diverse upstream stimuli.

### 3.4. Senescence and Ferroptosis: Expanding Functional Scope

Macrophage dysfunction extends beyond polarization. Senescent macrophages contribute to necrotic core expansion. Oxymatrine stabilizes SIRT1 protein, enhances p53 deacetylation, and delays macrophage senescence in ApoE^−^/^−^ mice [3]. Ferroptosis, an iron-dependent form of regulated cell death, is also regulated by SIRT1. Paclitaxel attenuates macrophage ferroptosis through activation of the SIRT1/Nrf2/GPX4 pathway [30], while inhibition of SIRT1 exacerbates ferroptotic lipid peroxidation [31]. These findings broaden the conceptual role of macrophage SIRT1 from inflammatory modulator to guardian of metabolic viability.

## 4. Vascular Smooth Muscle Cells: SIRT1 and the Control of Phenotypic Plasticity

### 4.1. VSMCs as Structural and Inflammatory Contributors to Plaque Evolution

Although macrophages dominate early lesion inflammation, vascular smooth muscle cells (VSMCs) ultimately determine plaque architecture and stability [32,33,34]. Traditionally viewed as passive matrix-producing cells, VSMCs are now recognized as highly plastic, capable of transitioning from a contractile phenotype to synthetic, inflammatory, osteogenic or macrophage-like states [1]. This phenotypic switching contributes to fibrous cap thinning, extracellular matrix remodeling and, in advanced lesions, plaque vulnerability. Because VSMCs are metabolically active and sensitive to oxidative stress, SIRT1-mediated regulation of mitochondrial function, redox balance and senescence may be particularly consequential in this cell type.

### 4.2. SIRT1 and VSMC Senescence

Cellular senescence in VSMCs promotes extracellular matrix degradation and inflammatory signaling, thereby destabilizing plaques. Zhou et al. demonstrated, using high-fat diet-fed ApoE^−^/^−^ mice and cultured VSMCs, that paeonol directly interacts with SIRT1 and activates the SIRT1/P53/TRF2 pathway [35]. Activation of this axis preserved telomere integrity, reduced senescence-associated β-galactosidase expression and attenuated lesion development. Mechanistically, SIRT1 deacetylates p53, reducing its transcriptional activity and thereby limiting cell-cycle arrest [1,30]. At the same time, SIRT1 stabilizes telomere-associated proteins such as TRF2, preserving chromosomal integrity [30]. This dual action positions SIRT1 as a critical regulator of genomic stability in VSMCs. Complementary evidence comes from naringenin-treated aged ApoE^−^/^−^ mice, in which enhanced SIRT1 activation increased FOXO3a and PGC-1α deacetylation, improved mitochondrial biogenesis and reduced markers of vascular senescence [36]. These findings indicate that SIRT1-mediated mitochondrial maintenance delays stress-induced premature senescence in VSMCs.

### 4.3. Mitochondrial Biogenesis and Energetic Integrity

Mitochondrial dysfunction has emerged as a central driver of vascular aging and VSMC phenotypic instability [37,38,39]. Sung et al. demonstrated that SRT1720, a pharmacological SIRT1 activator, restored mitochondrial function in oleic acid-treated VSMCs [40]. SIRT1-mediated deacetylation of PGC-1α enhanced mitochondrial biogenesis, reduced mtROS production and improved ATP generation. Importantly, inhibition of SIRT1 abrogated these effects, confirming pathway specificity. Jiawei Dachaihu decoction has also been shown to upregulate SIRT1/PGC-1α/TFAM signaling, restoring mitochondrial stability in atherosclerotic mice subjected to chronic stress [7]. This finding connects mitochondrial maintenance with both metabolic and psychosocial stress contexts. Together, these data support the view that SIRT1 safeguards mitochondrial integrity in VSMCs, thereby preserving contractile phenotype and plaque stability.

### 4.4. Microbiota–VSMC Axis: TMAO as a SIRT1 Suppressor

Emerging evidence links gut microbiota-derived metabolites to VSMC inflammatory programming [41,42]. Yin et al. demonstrated, using multi-omics analyses in AS mice, that elevated TMAO levels suppressed SIRT1 expression in VSMCs and promoted SM22α-mediated inflammatory signaling [20]. This effect was further supported by in vitro experiments showing that SIRT1 restoration attenuated TMAO-induced inflammatory gene expression. Zhou et al. further showed that TMAO suppressed AMPK activity, which secondarily reduced SIRT1 function and amplified ROS-mediated inflammation [19]. These findings suggest that SIRT1 operates as a metabolic effector translating microbial-derived nutrient signals into vascular inflammatory responses.

## 5. Aging and NAD^+^ Decline: SIRT1 in Vascular Longevity

### 5.1. Aging as the Dominant Risk Factor

Age is the strongest independent predictor of atherosclerosis [2]. With advancing age, NAD^+^ levels decline, mitochondrial function deteriorates and inflammatory tone increases, a phenomenon often described as “inflammaging.” Grootaert and Bennett synthesized extensive preclinical evidence demonstrating that reduced sirtuin activity accelerates vascular aging, whereas SIRT1 activation delays pathological remodeling [2]. This observation has led to the “NAD^+^–SIRT1–healthspan” hypothesis in vascular disease.

### 5.2. Mitochondrial-Targeted SIRT1 Activation

Karnewar et al. administered mitochondria-targeted esculetin to aged ApoE^−^/^−^ mice for prolonged periods and observed improved mitochondrial respiration, enhanced endothelial function and reduced plaque burden [43]. These benefits were associated with increased SIRT1 activation and reduced vascular senescence markers.

Importantly, esculetin not only activated SIRT1 but also modulated miRNA networks influencing PAI-1 expression and mitochondrial oxidative phosphorylation, suggesting that SIRT1-mediated vascular protection may involve multilayered transcriptional reprogramming.

### 5.3. NAD^+^ Dependency and Therapeutic Implications

Because SIRT1 enzymatic activity depends on NAD^+^ availability, strategies that increase NAD^+^ levels may indirectly enhance SIRT1 signaling. However, whether NAD^+^ supplementation alone is sufficient to meaningfully alter plaque biology remains uncertain. If NAD^+^ decline reflects chronic metabolic dysfunction rather than serving as a primary driver, simple restoration may not fully reverse disease progression. Thus, SIRT1 activation may need to be coupled with metabolic correction to achieve durable benefit.

## 6. Environmental Stressors and Circadian Regulation: SIRT1 as a Temporal Integrator

### 6.1. Environmental Pollutants and Oxidative Injury

Environmental exposures contribute significantly to cardiovascular risk [44,45]. Assempoor et al. demonstrated that endocrine-disrupting chemicals correlate with increased carotid intima-media thickness and plaque formation [46]. Although SIRT1 was not directly assessed in that meta-analysis, oxidative stress and inflammatory activation, both modulated by SIRT1—likely mediate these associations. Chen et al. provided more direct mechanistic insight by showing that acrolein exposure in ApoE^−^/^−^ mice suppressed AMPK/SIRT1 signaling and disrupted CLOCK/BMAL1 expression [43]. This resulted in increased inflammatory cytokine production and accelerated plaque formation.

### 6.2. Circadian Rhythm and Metabolic Coupling

Circadian gene disruption has been implicated in atherosclerosis progression. Because SIRT1 deacetylates and regulates CLOCK and BMAL1, it directly links cellular energy state to circadian oscillations [43]. Chen et al. further demonstrated that intermittent fasting restored AMPK activation, increased SIRT1 expression and normalized circadian gene expression in acrolein-exposed mice [43]. These findings highlight SIRT1 as a temporal regulator that coordinates metabolic state with circadian control.

### 6.3. Smoking and Hypoxia Signaling

Cigarette smoke extract induces endothelial dysfunction and autophagy. Wang et al. demonstrated that mulberroside A attenuated smoke-induced atherosclerosis in ApoE^−^/^−^ mice by restoring SIRT1 expression and suppressing HIF-1α signaling [47]. Because SIRT1 deacetylates HIF-1α, reducing hypoxia-driven inflammatory activation, these data suggest that SIRT1 integrates hypoxic stress responses with vascular inflammation.

## 7. Integration Across Cell Types: A Systems-Level View

When examined in isolation, SIRT1 appears to exert distinct protective effects within individual vascular cell types. However, when considered collectively across endothelial cells, macrophages and VSMCs, a broader and more coherent mechanistic pattern emerges: SIRT1 does not function as a linear pathway inhibitor but as a systems-level integrator of metabolic and inflammatory stress.

In endothelial cells, SIRT1 primarily operates as a redox stabilizer. By deacetylating eNOS and activating Nrf2-dependent antioxidant transcriptional programs, SIRT1 preserves nitric oxide bioavailability and suppresses ROS-driven NLRP3 inflammasome activation [17,18]. These actions collectively restrain the earliest steps of leukocyte adhesion and endothelial dysfunction, thereby limiting lesion initiation.

In macrophages, however, SIRT1 assumes a broader regulatory role that extends beyond oxidative control. Here, it modulates inflammatory polarization through NF-κB deacetylation, restrains excessive M1 activation, and promotes alternative M2 phenotypes via PPARγ signaling [27]. Simultaneously, SIRT1 preserves macrophage viability by delaying p53-driven senescence and suppressing ferroptotic cell death through Nrf2/GPX4-dependent mechanisms [30]. Thus, in macrophages, SIRT1 integrates inflammatory tone with metabolic fitness and cell survival.

Within VSMCs, SIRT1 shifts from an inflammatory regulator to a guardian of structural integrity. Through deacetylation of PGC-1α, SIRT1 promotes mitochondrial biogenesis and maintains energetic homeostasis [40]. Concurrent stabilization of telomere-associated proteins such as TRF2 limits replicative senescence and preserves contractile phenotype [35]. In this cellular context, SIRT1 directly influences fibrous cap thickness and plaque stability rather than inflammatory amplification.

Aging introduces yet another regulatory dimension. Declining NAD^+^ levels reduce SIRT1 enzymatic activity, thereby weakening redox defenses, mitochondrial function and genomic stability [2]. Long-term activation of SIRT1 in aged models restores mitochondrial respiration and attenuates vascular senescence [43], suggesting that SIRT1 links age-associated metabolic decline to structural deterioration of the arterial wall.

Under environmental stress conditions, including acrolein exposure and circadian disruption, SIRT1 further integrates metabolic and temporal signals [43,47]. By interacting with AMPK and circadian regulators such as CLOCK/BMAL1, SIRT1 couples nutrient availability and environmental cues to inflammatory output.

Taken together, these observations suggest that SIRT1 does not occupy a peripheral anti-inflammatory niche. Instead, it resides within a hierarchical signaling architecture in which AMPK and NAD^+^ availability act as upstream metabolic gatekeepers, SIRT1 serves as a central regulatory hub, and cell-type-specific transcriptional programs translate its activity into distinct functional outcomes. In this framework, suppression of SIRT1 represents not merely the loss of an anti-inflammatory enzyme, but the collapse of a metabolic buffering system that normally constrains vascular stress responses. Accordingly, SIRT1 should be conceptualized not as a single therapeutic target in isolation, but as a nodal regulator embedded within dynamic metabolic hierarchies that shape plaque evolution.

Emerging evidence suggests that, in addition to SIRT1, other members of the sirtuin family, particularly SIRT4, may contribute to vascular homeostasis through complementary mechanisms [48]. While SIRT1 primarily functions as a nuclear and cytosolic deacetylase regulating inflammatory signaling, oxidative stress, and endothelial function, SIRT4 is localized in mitochondria and modulates metabolic flux, including fatty acid oxidation and glutamine metabolism [49]. Recent studies indicate that SIRT4 may exert anti-atherosclerotic effects by limiting mitochondrial oxidative stress and suppressing inflammatory signaling pathways [50]. Notably, loss of SIRT4 has been associated with enhanced NF-κB activation and increased chemokine expression, promoting vascular inflammation and leukocyte recruitment [50]. These observations suggest that SIRT1 and SIRT4 may form a coordinated regulatory axis linking cellular metabolism with inflammatory control. While SIRT1 integrates upstream metabolic cues such as NAD^+^ availability and AMPK signaling to regulate transcriptional programs, SIRT4 complements this function by maintaining mitochondrial metabolic integrity [49]. Together, these dual-layer regulations may provide a more comprehensive protective network against atherosclerosis progression. However, the precise interplay between SIRT1 and SIRT4 in vascular cells remains incompletely understood and warrants further investigation.

## 8. Human Evidence: Translating SIRT1 Biology into Clinical Context

Although preclinical studies consistently demonstrate protective roles of SIRT1 activation, human evidence remains comparatively nuanced. Analyses of carotid endarterectomy specimens have reported reduced SIRT1 expression in unstable plaques, particularly in endothelial and macrophage-rich regions characterized by elevated oxidative stress and inflammatory cytokine production. Reduced SIRT1 levels correlate with increased NF-κB activation and impaired nitric oxide bioavailability, supporting its pathophysiological relevance in human lesions. However, context-dependent variability has also been observed. In certain vascular pathologies, including aneurysmal tissues, SIRT1 expression may be upregulated, possibly reflecting a compensatory adaptive response rather than uniform downregulation. These findings suggest that SIRT1 expression in human disease is dynamic and may represent an adaptive stress response whose protective capacity becomes insufficient under sustained metabolic burden. This complexity highlights the need for spatially resolved and activity-based measurements of SIRT1 within human plaques rather than reliance on expression levels alone. Rum et al. analyzed SIRT1 expression in ascending aortic tissues obtained from patients with aneurysm and dissection [51]. Interestingly, they observed altered SIRT1 levels depending on pathological context, with some diseased tissues exhibiting compensatory upregulation. In contrast, systemic atherosclerotic disease has generally been associated with reduced SIRT1 expression. This apparent discrepancy suggests that SIRT1 expression in human vascular tissue may reflect adaptive stress responses rather than uniform protective downregulation.

Moreover, metabolic phenotypes linked to subclinical atherosclerosis may intersect with SIRT1 regulation. Hypertriglyceridemic waist phenotype, for example, has been strongly associated with increased atherosclerotic burden in population studies [52]. Given the central role of SIRT1 in lipid metabolism and PPARγ signaling [29], metabolic dysregulation may indirectly impair SIRT1-mediated vascular protection. In the context of diabetes, which markedly accelerates atherosclerosis, SIRT1 dysregulation has also been implicated. Hyperglycemia enhances neutrophil extracellular trap (NET) formation via dysfunctional SIRT1–PAD4 interactions [53], linking impaired SIRT1 signaling to exaggerated inflammatory responses in diabetic vasculature. Such findings expand the relevance of SIRT1 beyond macrophage and endothelial biology to innate immune dysregulation in metabolic disease.

Despite these associations, direct quantification of SIRT1 activity within human coronary plaques remains sparse. High-resolution imaging modalities such as intravascular ultrasound and optical coherence tomography demonstrate plaque stabilization with lipid-lowering therapies [5], yet whether SIRT1 signaling correlates with plaque regression or cap thickening in humans has not been rigorously examined. Thus, while preclinical evidence is compelling, translational validation remains incomplete.

Beyond its mechanistic role in vascular biology, SIRT1 has attracted increasing interest as both a potential biomarker of metabolic resilience and a therapeutic target in cardiometabolic disease. Reduced SIRT1 activity has been associated with metabolic syndrome, diabetes, and heightened inflammatory burden, suggesting that circulating or tissue-level SIRT1 activity may reflect systemic metabolic status and cardiovascular risk. Indeed, SIRT1 expression in peripheral blood mononuclear cells has been linked to inflammatory burden and adverse cardiovascular risk profiles. However, substantial variability across metabolic states complicates its predictive utility, and whether SIRT1 activity can reliably stratify patients who may benefit from NAD^+^-restoring interventions or metabolic therapies remains uncertain. From a therapeutic perspective, modulation of SIRT1 signaling could complement conventional lipid-lowering strategies by targeting residual inflammatory and metabolic risk. For instance, pharmacological approaches that enhance NAD^+^ availability or activate AMPK signaling may indirectly augment SIRT1 activity and improve vascular metabolic homeostasis. Future clinical studies are therefore needed to longitudinally evaluate SIRT1 activity in relation to plaque progression, cardiovascular outcomes, and treatment response.

These findings collectively suggest that although SIRT1 signaling is consistently protective in experimental systems, its activity in human atherosclerotic plaques likely reflects a dynamic balance between metabolic stress and adaptive vascular responses.

## 9. SIRT1 and Lipid-Lowering Therapy: Complementary or Redundant?

Statins and PCSK9 inhibitors reduce LDL cholesterol and promote plaque stabilization [5]. However, substantial residual inflammatory risk persists, as evidenced by clinical trials targeting IL-1β and other inflammatory mediators. SIRT1 intersects with lipid metabolism through regulation of PPARγ and cholesterol efflux pathways [22,29]. For example, HAND2-AS1-mediated enhancement of SIRT1 expression promotes ABCA1/G1-dependent cholesterol efflux in macrophages [22]. Similarly, lincRNA-p21 modulates SIRT1/Pcsk9 signaling, influencing lipid deposition in ApoE^−^/^−^ mice [23]. These observations suggest that SIRT1 activation may complement lipid-lowering therapy by addressing residual inflammatory and metabolic dysregulation rather than replacing LDL-targeted interventions. However, whether SIRT1 activation provides an additive benefit in the presence of optimal lipid control remains untested in large clinical settings.

## 10. Systems Pharmacology: Convergence of Diverse Agents on SIRT1

A striking theme across the literature is that chemically diverse compounds converge upon SIRT1 signaling. Flavonoids such as dihydromyricetin [28], natural products such as oxymatrine [3], herbal formulations such as Nao-Xin-Qing tablets [11], saponins [29], and even vitamin D [8] all exert vascular protective effects through SIRT1-dependent mechanisms. Hydroxytyrosol and other nutraceuticals similarly activate SIRT1 and attenuate oxidative stress pathways in vascular cells [12]. In inflammatory contexts, SIRT1 activation suppresses NLRP3 inflammasome signaling [26] and regulates mTORC2-mediated immune responses [8]. Even in non-classical models, such as cobalt–chromium particle exposure, inhibition of the SIRT1/Nrf2/GPX4 pathway induces ferroptosis and inflammatory amplification [31], reinforcing SIRT1’s role as a stress-buffering hub. This pharmacological convergence suggests that SIRT1 functions as a nodal integrator within vascular stress networks. However, it also raises concerns about specificity: because SIRT1 lies downstream of AMPK and NAD^+^ availability, upstream metabolic correction may be necessary for sustained therapeutic benefit.

## 11. Precision Cardiovascular Medicine: Targeting Context Rather than Enzyme Alone

Recent advances in multi-omics and precision cardiology emphasize the heterogeneity of atherosclerotic lesions [54]. Single-cell transcriptomic analyses reveal distinct endothelial, macrophage and VSMC subpopulations within plaques, each with unique metabolic signatures. Within this heterogeneous environment, SIRT1 activity likely varies spatially and temporally. For instance, early lesions characterized by oxidative stress may benefit from SIRT1-mediated antioxidant activation [17,18], whereas advanced necrotic cores dominated by ferroptosis may require SIRT1-driven GPX4 stabilization [30]. Thus, indiscriminate systemic activation of SIRT1 may not achieve uniform benefit. Instead, cell-type-specific modulation, potentially through targeted nanoparticles, RNA-based delivery systems or gene-editing technologies, may allow precise engagement of SIRT1 where it is most protective. In this regard, small activating RNA strategies targeting SIRT1 have shown promise in fibrotic models [55], raising the possibility of vascular-specific gene activation approaches in the future.

SIRT1 exerts regulatory functions across multiple organ systems, including liver glucose metabolism, adipose tissue lipolysis, and central energy balance. Excessive systemic activation may theoretically enhance hepatic gluconeogenesis, alter appetite regulation, or disrupt endocrine homeostasis. Furthermore, SIRT1 interacts with circadian regulators and hypoxia signaling pathways, raising concerns regarding unintended temporal or metabolic perturbations. These considerations underscore the necessity for spatially restricted therapeutic approaches. Nanoparticle-based delivery systems, macrophage-targeted liposomes, endothelium-specific peptides, and RNA-based gene-activating platforms represent promising strategies for selectively enhancing SIRT1 activity within atherosclerotic lesions while minimizing systemic effects. Precision targeting may therefore be essential for safe clinical translation.

## 12. Controversies and Unresolved Questions

A critical conceptual question is whether SIRT1 functions as a primary driver of vascular protection or as a downstream adaptive stress responder. In many contexts, SIRT1 activation follows AMPK signaling and NAD^+^ restoration, positioning it downstream of broader metabolic regulatory hierarchies. Oxidative stress, nutrient deprivation, and mitochondrial dysfunction frequently induce compensatory SIRT1 upregulation as part of cellular stress adaptation programs. This hierarchical positioning suggests that SIRT1 may act as a metabolic “shock absorber,” buffering cellular injury rather than independently initiating protective signaling. Consequently, pharmacological activation of SIRT1 alone may not fully recapitulate the coordinated upstream metabolic adaptations required for durable vascular protection. Future therapeutic strategies may therefore need to target metabolic context rather than SIRT1 in isolation.

Despite extensive preclinical data, several key uncertainties remain. First, causality versus compensation: Is reduced SIRT1 activity a primary driver of lesion progression, or does it reflect metabolic exhaustion secondary to chronic inflammation? Observations of context-dependent SIRT1 expression in human tissues [51] suggest adaptive complexity. Second, NAD^+^ dependency: Because SIRT1 requires NAD^+^ for enzymatic activity, declining NAD^+^ levels with aging may limit pharmacological efficacy [2]. Whether NAD^+^ supplementation meaningfully enhances vascular SIRT1 activity remains uncertain. Third, stage specificity: Early endothelial dysfunction and late plaque instability involve distinct cellular processes. It is conceivable that SIRT1 activation confers maximal benefit during early inflammatory phases, but less impact once advanced necrotic cores have formed. Fourth, pathway redundancy: Inflammatory signaling networks are highly redundant. Even if SIRT1 suppresses NF-κB activation, parallel inflammatory pathways may compensate. Fifth, systemic effects: Because SIRT1 regulates metabolism across multiple organs, systemic activation may produce unintended metabolic consequences. Addressing these questions will require carefully designed mechanistic and translational studies.

In addition, several methodological limitations should be acknowledged. First, many studies rely on pharmacological activators such as resveratrol or SRT1720, which may exert off-target effects independent of SIRT1 activation. Consequently, attributing observed vascular benefits exclusively to SIRT1 signaling may oversimplify the underlying mechanisms. Second, genetic knockout models often involve lifelong deletion of SIRT1, which may trigger developmental compensatory mechanisms that do not reflect the dynamics of adult disease progression. Moreover, most mechanistic studies utilize ApoE^−^/^−^ or LDLR^−^/^−^ murine models that only partially reproduce the complexity and heterogeneity of human atherosclerotic plaques. Finally, measurement of SIRT1 expression levels is frequently used as a surrogate for enzymatic activity. However, because SIRT1 activity is strongly dependent on intracellular NAD^+^ availability, expression levels alone may not accurately represent functional signaling capacity. These methodological constraints highlight the need for more precise approaches to assess SIRT1 activity in human vascular tissues.

Several important knowledge gaps remain. First, the spatial and temporal dynamics of SIRT1 activity within human atherosclerotic plaques are poorly characterized. Whether SIRT1 activity differs between early lesions, fibrous plaques, and rupture-prone necrotic cores remains unclear. Second, the hierarchical relationship between SIRT1 and upstream metabolic regulators such as AMPK and NAD^+^ metabolism requires further clarification. Determining whether SIRT1 functions primarily as a driver of vascular protection or as a downstream adaptive stress responder remains a key unresolved issue. Third, the heterogeneity of vascular cell populations identified through single-cell transcriptomics raises the possibility that SIRT1 activity may vary significantly among endothelial, immune, and smooth muscle subpopulations within plaques. Understanding these context-dependent effects will be essential for developing targeted therapeutic strategies.

## 13. Final Synthesis: SIRT1 as a Metabolic-Inflammatory Integrator

Across endothelial cells, macrophages, VSMCs, aging models and environmental contexts, a consistent pattern emerges: SIRT1 integrates metabolic sensing with inflammatory restraint. It suppresses ROS-dependent inflammasome activation in endothelial cells [17,18], regulates macrophage polarization and ferroptosis [27,30], preserves mitochondrial function in VSMCs [35,40], delays vascular aging [36], and integrates circadian regulation with environmental stress responses [43,47]. Rather than functioning as a single anti-inflammatory enzyme, SIRT1 acts as a metabolic-inflammatory rheostat embedded within hierarchical signaling networks governed by AMPK, NAD^+^ availability and nutrient-derived metabolites [19,20]. The translational challenge lies not in proving that SIRT1 influences vascular biology—that is well established—but in identifying when, where and how to modulate it most effectively within the heterogeneous landscape of human atherosclerosis. In this sense, SIRT1 may ultimately prove most valuable not as a universal therapeutic target, but as a precision node whose activation must be contextually tailored to disease stage, metabolic status and cellular microenvironment.

## 14. Conclusions

Atherosclerosis represents the cumulative outcome of sustained metabolic stress, maladaptive inflammation, mitochondrial dysfunction and age-related decline in vascular resilience. Across this complex landscape, SIRT1 emerges not as a single-pathway inhibitor, but as a metabolic–inflammatory integrator embedded within hierarchical regulatory networks that coordinate cellular stress responses.

**Figure 1:**
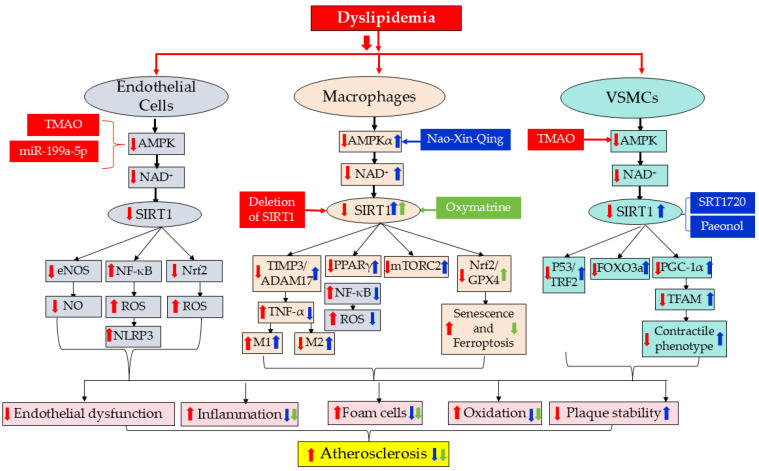
SIRT1-centered regulatory network linking metabolic stress to vascular cell dysfunction in atherosclerosis. Dyslipidemia acts as a primary metabolic trigger that drives pathological responses in multiple vascular cell types, including endothelial cells, macrophages, and vascular smooth muscle cells (VSMCs). In endothelial cells, dyslipidemia-associated factors such as trimethylamine N-oxide (TMAO) and miR-199a-5p suppress AMPK activity and reduce intracellular NAD^+^ levels, leading to decreased SIRT1 activity. Reduced SIRT1 signaling subsequently impairs endothelial nitric oxide synthase (eNOS) activation, enhances NF-κB–mediated inflammatory signaling, and increases oxidative stress through Nrf2-dependent pathways, ultimately promoting NLRP3 inflammasome activation and endothelial dysfunction. In macrophages, decreased SIRT1 activity, either through genetic deletion or metabolic suppression, disrupts several regulatory pathways, including the TIMP3/ADAM17 axis, NF-κB signaling, and PPARγ-mediated macrophage polarization. These changes enhance TNF-α production, increase reactive oxygen species (ROS), promote pro-inflammatory M1 polarization, and reduce anti-inflammatory M2 macrophages. Additionally, SIRT1 deficiency alters Nrf2/GPX4 and mTORC2 signaling, contributing to cellular senescence and ferroptosis. Pharmacological agents such as oxymatrine and Nao-Xin-Qing can partially restore SIRT1 signaling and mitigate macrophage inflammatory activation. In VSMCs, TMAO-mediated inhibition of AMPK reduces NAD^+^ levels and suppresses SIRT1 activity. Downstream effects include dysregulation of P53/TRF2, FOXO3a, and PGC-1α signaling pathways, leading to impaired mitochondrial biogenesis, reduced TFAM expression, and loss of the contractile phenotype. Pharmacological activation of SIRT1 by compounds such as SRT1720 and paeonol can counteract these changes. Collectively, dysregulation of SIRT1 signaling across endothelial cells, macrophages, and VSMCs contributes to endothelial dysfunction, vascular inflammation, foam cell formation, oxidative stress, and reduced plaque stability, ultimately accelerating the progression of atherosclerosis. Red arrows indicate pro-atherosclerotic effects, whereas blue and green arrows indicate anti-atherosclerotic effects. Upward arrows denote increased activity or expression, while downward arrows denote decreased activity or suppression.

Evidence from endothelial cells, macrophages and vascular smooth muscle cells collectively indicates that SIRT1 maintains vascular homeostasis through cell-type-specific mechanisms. In endothelial cells, it stabilizes redox balance and preserves nitric oxide bioavailability. In macrophages, it modulates inflammatory polarization, limits senescence and restrains ferroptotic cell death. In vascular smooth muscle cells, it preserves mitochondrial integrity and genomic stability, thereby supporting fibrous cap maintenance. These coordinated actions converge on a shared outcome: attenuation of plaque progression and enhancement of structural stability (Figure 1).

However, the protective capacity of SIRT1 is neither absolute nor autonomous. Its activity is constrained by upstream metabolic gatekeepers, including AMPK signaling and NAD^+^ availability, both of which decline under conditions of aging, metabolic syndrome and environmental stress. Thus, SIRT1 functions less as an independent therapeutic lever and more as a nodal rheostat whose efficacy depends on broader metabolic context.

The translational challenge, therefore, is not simply to activate SIRT1, but to define when, where and under which metabolic conditions such activation will meaningfully reshape plaque biology. Precision modulation—integrating NAD^+^ restoration, metabolic correction, circadian alignment and cell-specific targeting—may be required to unlock its therapeutic potential.

In this light, SIRT1 should be conceptualized as a systems-level regulator of vascular stress adaptation. Future investigations must move beyond reductionist activation strategies and instead delineate the temporal, spatial and metabolic hierarchies that determine SIRT1 responsiveness in human atherosclerosis. Only through such integrative approaches can SIRT1 transition from a compelling mechanistic target to a clinically transformative node in precision cardiovascular medicine.

## Data Availability

No new data were created or analyzed in this study. Data sharing is not applicable to this article.
